# Hierarchical Development of Early Visual-Spatial Abilities – A Taxonomy Based Assessment Using the MaGrid App

**DOI:** 10.3389/fpsyg.2020.00871

**Published:** 2020-05-20

**Authors:** Stefanie Jung, Anna Meinhardt, David Braeuning, Stephanie Roesch, Véronique Cornu, Tahereh Pazouki, Christine Schiltz, Jan Lonnemann, Korbinian Moeller

**Affiliations:** ^1^Leibniz-Institut für Wissensmedien, Tübingen, Germany; ^2^Department of Psychology, Eberhard Karls University Tübingen, Tübingen, Germany; ^3^DIPF Leibniz Institute for Research and Information in Education, Frankfurt, Germany; ^4^Center for Individual Development and Adaptive Education of Children at Risk (IDeA), Frankfurt, Germany; ^5^LEAD Graduate School and Research Network, University of Tübingen, Tübingen, Germany; ^6^Hector Research Institute of Education Sciences and Psychology, University of Tübingen, Tübingen, Germany; ^7^Luxembourg Centre for Educational Testing (LUCET), University of Luxembourg, Esch-sur-Alzette, Luxembourg; ^8^Department of Behavioral and Cognitive Sciences (DBCS), University of Luxembourg, Esch-sur-Alzette, Luxembourg; ^9^Empirical Childhood Research, University of Potsdam, Potsdam, Germany; ^10^Centre for Mathematical Cognition, School of Science, Loughborough University, Loughborough, United Kingdom

**Keywords:** visual-spatial abilities, 2 by 2 taxonomy, geometry, tablet-based approach, MaGrid

## Abstract

Visual-spatial abilities (VSA) are considered a building block of early numerical development. They are intuitively acquired in early childhood and differentiate in further development. However, when children enter school, there already are considerable individual differences in children’s visual-spatial and numerical abilities. To better understand this diversity, it is necessary to empirically evaluate the development as well as the latent structure of early VSA as proposed by the 2 by 2 taxonomy of Newcombe and Shipley (2015). In the present study, we report on a tablet-based assessment of VSA using the digital application (app) MaGrid in kindergarten children aged 4–6 years. We investigated whether the visual-spatial tasks implemented in MaGrid are sensitive to replicate previously observed age differences in VSA and thus a hierarchical development of VSA. Additionally, we evaluated whether the selected tasks conform to the taxonomy of VSA by Newcombe and Shipley (2015) applying a confirmatory factor analysis (CFA) approach. Our results indicated that the hierarchical development of VSA can be measured using MaGrid. Furthermore, the CFA substantiated the hypothesized factor structure of VSA in line with the dimensions proposed in the taxonomy of Newcombe and Shipley (2015). Taken together, the present results advance our knowledge to the (hierarchical) development as well as the latent structure of early VSA in kindergarten children.

## Introduction

Early numerical development was suggested to build on both spatial-geometric and numerical-quantitative concepts and the acquisition of corresponding abilities (Sarama and Clements, 2004; Jirout and Newcombe, 2015; Newcombe et al., 2015). These skills were argued to be acquired intuitively in early childhood (e.g., Newcombe et al., 2015), but their close association persists in adulthood (Dehaene et al., 1999; Hubbard et al., 2005).

However, already at the age of kindergarten, there are large individual differences in children’s spatial and numerical skills (Krajewski and Schneider, 2009; Newcombe and Frick, 2010), which also have long-term consequences: For example, longitudinal studies revealed that children’s spatial as well as basic numerical abilities at the age of kindergarten predict their mathematical achievement in primary school and beyond (Duncan et al., 2007; Krajewski and Schneider, 2009; Verdine et al., 2017). More recent evidence from large-scale factor analytic studies suggested strong relations among visual-spatial and mathematic skills in first, third and sixth graders (Mix et al., 2016, 2017).

Visual-spatial abilities (VSA), in particular, are an important building block when it comes to acquiring geometric abilities (Franke and Reinhold, 2007), indicating that their impact goes beyond typically considered basic numerical abilities such as counting and magnitude understanding (cf. Clements, 1998). However, there are multiple abilities summarized under the broad umbrella of VSA for which it is difficult to specify theoretical concepts associated with this term (Eliot and Smith, 1983; Carroll, 1993; Newcombe et al., 2015; Mix et al., 2016). Only recently, Newcombe and Shipley (2015) proposed a top-down systematic taxonomy of VSA, which considers and integrates prior distinctions of different dimensions of VSA. This taxonomy defines VSA along two dimensions: first, VSA being either intrinsic to vs. extrinsic between objects (following the neural organization of spatial thinking, e.g., Chatterjee, 2008). Second, VSA being related to static vs. dynamic aspects of objects (considering propositions by e.g., Kozhevnikov et al., 2002). Such a systematic attempt to define the actual nature of VSA and to understand their latent cognitive components may provide a promising framework based on which VSA can be assessed and promoted.

In the present paper, we aimed at validating the 2 by 2 taxonomy of Newcombe and Shipley (2015) using an assessment procedure for VSA in kindergarten children aged 4 to 6 years from both a theoretical and a behavioral perspective. From a theoretical perspective, we investigated how VSA develop with respect to the intrinsic-extrinsic dimension as well as to the static-dynamic dimension as proposed in the 2 by 2 taxonomy of VSA. From a behavioral perspective, we investigated the hierarchical development of VSA as assessed by the digital application (app) MaGrid (“Math on Grid”; Cornu et al., 2017; Pazouki et al., 2018). In the following, we will first report on recent approaches to theoretically categorize VSA before we consider their hierarchical development. Subsequently, we introduce the tablet-based app MaGrid to provide an idea of its functionality and how the app is currently used to promote VSA.

### A Taxonomy of Visual-Spatial Abilities

A comprehensive understanding of VSA, which are generally referred “to skill[s] in representing, transforming, generating, and recalling symbolic, non-linguistic information” (Linn and Petersen, 1985, p. 1,482), is essential to develop valid assessment and training tools. However, its complexity has long hampered a coherent definition. Still today, there are inconsistencies and contradictions in the literature on VSA. Although different bottom-up factor-analytical approaches have confirmed the variety of spatial abilities (Newcombe and Shipley, 2015), they did not lead to a consensus on the definition of this term.

Uttal et al. (2013) were among the first to adopt an opposing top-down approach: they worked on the development of a two-dimensional classification system of VSA. This classification system is referred to by the 2 by 2 taxonomy proposed by Newcombe and Shipley (2015) and incorporates evidence from cognitive, linguistic and neural findings (Palmer, 1978; Talmy, 2000; Chatterjee, 2008). Within this taxonomy, four different categories of VSA are defined: Intrinsic-static (i.e., perceiving objects), intrinsic-dynamic (i.e., assembling small units into larger ones, mental rotation), extrinsic-static (i.e., understanding abstract spatial concepts), and extrinsic-dynamic (i.e., perspective taking) VSA.

Intrinsic processes require only consideration of the object at hand, whereas object surroundings in terms of a reference frame are not considered. A reference frame is understood as a coordinate system needed to determine the position of an object in space in relation to others from a certain perspective (Talmy, 2000). Extrinsic processes, in contrast, involve relations between different objects as well as the spatial configuration of objects within a reference frame. Static and dynamic aspects of single or multiple objects concern the immobility or motion of objects. On the one hand, an object can remain static, which means that it does not change its position, orientation, and/or dimension. On the other hand, objects can be manipulated physically or mentally, which involves changes in position and orientation. This manipulation defines dynamic VSA. For example, the picture of a car can be viewed as a 2D-static object. The car itself, however, can also be viewed as a 3D dynamic object. In 3D, the car can be rotated or moved. It is also possible to take, for instance, the perspective of its driver.

Literature on VSA provides considerable support for the 2 by 2 taxonomy of Newcombe and Shipley (2015, e.g., Newcombe, 2018, for a review). It is therefore increasingly used as a theoretical framework for the classification of VSA. For example, Hodgkiss et al. (2018) tested VSA of 7- to 11-years-old children using five different tasks, which the authors assigned to the four categories of VSA according to the 2 by 2 taxonomy (i.e., intrinsic-static: visual embedding; intrinsic-dynamic: mental rotation and mental folding; extrinsic-static: spatial scaling; extrinsic-dynamic: photo spatial perspective taking). They observed that task performance differed significantly between categories. Interestingly, only intrinsic-dynamic and extrinsic-static VSA were found to predict performance in STEM subjects (e.g., biology, chemistry, physics). However, while this provides evidence corroborating the taxonomy of Newcombe and Shipley (2015) the findings of Hodgkiss et al. (2018) do not yet reflect a validation of the taxonomy. To do so, it would be necessary to include more than one task per category of VSA and to evaluate the relations within vs. between tasks and categories, which the authors did only for intrinsic-dynamic VSA.

In contrast, Mix et al. (2018) assessed two tasks per category of the 2 by 2 taxonomy in a *post hoc* analysis of previously published data (Mix et al., 2017). However, their findings did not support the validity of the theoretically assumed 2 by 2 structure of VSA. Using a confirmatory factor analysis (CFA) approach on data of school children (i.e., first, third and sixth grade), the authors did not observe evidence for an overall 2 by 2 structure. Instead, their CFA results showed that the static-dynamic 2-factor model did not provide a better fit than a single factor model. Consequently, there was no differentiation along the static-dynamic dimension of VSA. Furthermore, the differentiation between intrinsic and extrinsic VSA was substantiated by the CFA, but only for first and third graders. For sixth graders, a single factor model was found to fit the data best. Based on these findings, Mix et al. (2018) suggested that the latent structure of VSA may change over the course of their development. They proposed to further investigate the developmental trajectories of VSA which was one aim of the present study.

### Hierarchical Development of VSA Considering the 2 by 2 Taxonomy

Studies on the early development of VSA demonstrated that these abilities begin to develop already in infancy and further evolve during childhood (Frick and Wang, 2014). From the literature, it is reasonable to assume that this hierarchical development of VSA may also be reflected in the 2 by 2 taxonomy of Newcombe and Shipley (2015) although the complexity involved in categorizing VSA and tasks can hardly be captured by such an approach (Newcombe, 2018).

In the course of development, it is assumed that the development of intrinsic VSA precedes the development of extrinsic VSA (Newcombe and Huttenlocher, 2006). Similarly, the development of static VSA is assumed to precede the development of dynamic VSA (Okamoto et al., 2015). In particular and concerning the intrinsic-static category, Clements (1998), for example, analyzed the characteristics by which 3–6 years old kindergarten children distinguish between different shapes (e.g., circles and rectangles). The authors observed that almost all children were able to recognize and externally verbalize the object’s characteristics. However, they also found that object recognition did improve with age.

Similar results were reported by Stiles and Tada (1996) who assessed how kindergarten children of different age groups (i.e., 3–3.5, 3.5–4, 4–4.5, and 4.5–5 years) segmented objects (e.g., +, ×, ^∗^) into parts or integrated parts to objects. The authors found that younger children segmented forms into more components than older children, because they perceived lines as discontinuous due to, for instance, an intersection at the midpoint. Older children, instead, perceived the lines as continuous across such an intersection. This indicates that they already seem to have acquired more elaborate shape recognition skills and thus a more abstract representation of the respective object.

Based on such an abstract representation of forms and objects (e.g., length and distance of lines, or angles; Lee et al., 2012), children may then develop extrinsic-static abilities that involve an understanding of spatial relations between objects and the environment as well as the size and scaling of objects. Then again, processing of extrinsic-static information improves with age and individual experiences (Newcombe and Huttenlocher, 2006; see also Okamoto et al., 2015, for an overview).

In contrast to the understanding of intrinsic-static or extrinsic-static characteristics of objects, dynamic VSA often involve transforming, (mentally) rotating, or assembling (a set of) objects as well as perspective taking (Uttal et al., 2013; Newcombe and Shipley, 2015). With regard to the intrinsic-dynamic category, Clements et al. (2004) investigated the development of this VSA in 3–7 years old children in a composition task of geometric figures. The successful development of intrinsic-dynamic VSA is seen as a prerequisite to cope with extrinsic-dynamic visual-spatial processing because extrinsic-dynamic VSA involve recognition of changing spatial relations of objects while considering the environment from different perspectives. Thereby, they involve self-to-object (i.e., perspective taking) and object-to-object (i.e., location learning) navigation (Okamoto et al., 2015), which develop throughout the early years of childhood.

**FIGURE 1:**
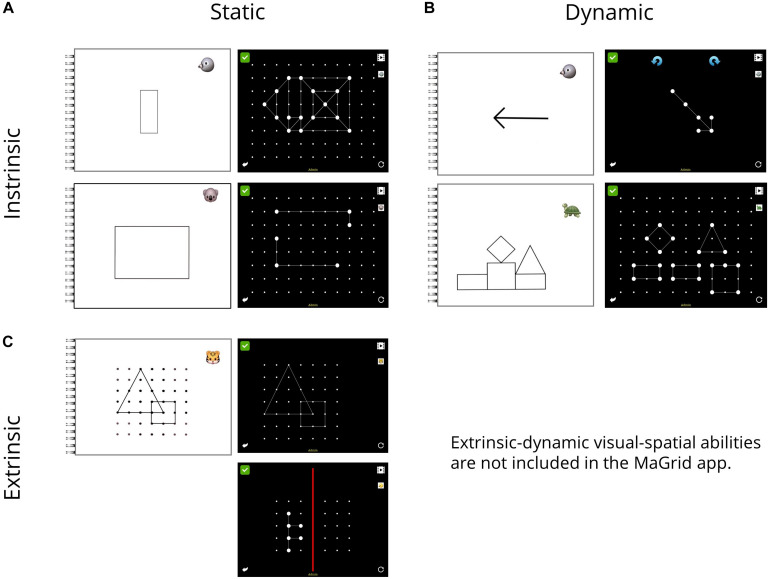
**(A–C)** Classification of MaGrid tasks in the taxonomy proposed by Newcombe and Shipley (2015). **(A)** Depicts an example of an item of the *Find forms* task in the top row and an item of the *Close form* task in the bottom row. For both tasks, the tablet display is shown on the right side and the corresponding booklet picture on the left side of the panel. **(B)** Gives an example of an item of the *Rotation* task in the top row and an item of the *Tangram* task on the bottom row. For both tasks, target objects are given in the booklet (left). **(C)** Shows an item of the *Reproduce forms I* task for which again a booklet is needed (left). The bottom row depicts an example of the *Reproduce forms II* task. Only the tablet is needed for this task. Tasks assessing extrinsic-dynamic VSA are not provided in the MaGrid app.

Despite the consideration of the different dimensions of VSA, the development of VSA along the intrinsic-extrinsic dimension cannot be assumed to be distinct from the development of VSA along the static-dynamic dimension. More likely, a development across both dimensions can be assumed. To be more specific, when considering the four categories of VSA as a 2 × 2 matrix (see also Figure 1), developmental trajectories would be expected both in the horizontal direction along the static-dynamic dimension as well as in the vertical direction along the intrinsic-extrinsic dimension. Consequently, intrinsic-static VSA are assumed to develop earlier than intrinsic-dynamic VSA while they also develop earlier than extrinsic-static VSA. Accordingly, within a specific age group, intrinsic-static VSA should be further developed than intrinsic-dynamic VSA, which should be more pronounced than extrinsic-static VSA and these again further developed than extrinsic-dynamic VSA (i.e., intrinsic-static > intrinsic-dynamic > extrinsic-static > extrinsic-dynamic). Based on this assumption, the 2 × 2 taxonomy of VSA by Newcombe and Shipley (2015) provides a framework not only for the structure of VSA but also for the development of VSA with age (Uttal et al., 2013; for the malleability of VSA).

Latest developments in digital technologies are influencing the development of assessment and training tools for VSA at an incredible speed, providing small and ready to use devices such as touch-operated smartphones and tablet devices. Tablets, in particular, are increasingly used in educational settings (e.g., Goodwin, 2012; Pazouki et al., 2018). Tablet-based trainings have been shown to improve VSA – even though these improvements have been found to differ from improvements gained in paper-based trainings (e.g., Lowrie et al., 2014, but see Lowrie et al., 2017), for partly contradictory results).

There is, however, no requirement for scientific validation for apps marketed as educational (Hirsh-Pasek et al., 2015). This is problematic for educators and parents alike when they want to ensure that children are using appropriate and effective apps for educational purposes (Hirsh-Pasek et al., 2015). In turn, this emphasizes the need for research and development of validated educational apps.

From the perspective of an app, tablets already seem to be attractive to young children as they encourage kindergarten children to become more closely and effectively involved in digital activities (Zaranis and Valla, 2017). And even very young children seem to be able to use tablets, as recently shown by Marsh et al. (2015). The authors observed that more than fifty percent of children between 0 and 5 years of age were able to drag objects on a tablet and follow shapes with their fingers on their own.

From an educational and scientific perspective, tablets seem suitable as they have been found to be effective for training and assessment of different cognitive abilities (e.g., Lowrie et al., 2014; Cornu et al., 2017). In this context, it is of particular importance that these apps consider the limited but developing, cognitive and motor skills of young children (Vatavu et al., 2015) as well as educational design principles to ensure learning (e.g., Cayton-Hodges et al., 2015). Taken together, these findings show the large potential of tablets used in education, even for young children, but also the need for the development of validated apps.

### MaGrid – A Tablet-Based Early Visual-Spatial and Mathematical Training

The recently introduced tablet-based training tool MaGrid for VSA and early numerical abilities (Cornu et al., 2017; Pazouki et al., 2018) aims at meeting this challenge. MaGrid training tasks are based on established developmental models of numerical cognition (Von Aster and Shalev, 2007) as well as further findings from empirical research on visual-spatial development. Thus, they line up with the few existing digital programs for training numerical skills, which are based on generally accepted theoretical concepts and scientific evidence (e.g., “Math Garden”; Straatemeier, 2014; “Math Shelf”; Schacter et al., 2016).

MaGrid is a tablet-based app for training building blocks of early numerical abilities. It provides a wide range of training tasks (i.e., 32 number specific and simple arithmetic tasks and 16 different visual-spatial tasks). These tasks target different aspects of visual-spatial (e.g., spatial perception, (mental) rotation, spatial visualization, and visual-motor integration) and related number-specific knowledge mostly at the preschool level for children aged 4–7 years. A novelty of MaGrid is its independence of any language instructions such as text or voice-overs, which is unique so far. In addition, MaGrid combines all the advantages of computer-based training tools. It allows user-friendly easy to administer individual learning in an interactive way and provides real-time feedback. The built-in logging- and monitoring-system allows to keep track of a children’s learning progress and to observe potential training-related improvements over time (Pazouki et al., 2018).

The effectiveness of MaGrid was evaluated empirically for kindergarten children (Cornu et al., 2017). In their intervention study, Cornu et al. (2017) realized a MaGrid training of VSA twice a week over a period of 10 weeks. The authors used various tasks such as *Find forms, Copy forms, Tangram, Rotation, Reproduce forms I* and *Reproduce forms II* (these tasks are used in the present study related to the 2 by 2 taxonomy of VSA), as well as *Find the pair, Figure completion, Odd-one out, Row completion, Line bisection, Figure bisection*, and *Symmetry* among others. Training effects were compared to a control group of kindergarten children (i.e., business-as-usual classroom following the Luxemburgish curriculum for kindergartens) who did not use the app. Results indicated that children who were trained with MaGrid significantly improved in some VSA (e.g., spatial orientation and visuo-motor integration) over the course of training. However, improvements in VSA were limited to the trained visual-spatial domains. The authors did not observe generalization to non-trained VSA or numerical skills. Nevertheless, this study showed the suitability of MaGrid for training VSA in kindergarten children. However, MaGrid has not yet been used as a tool for targeted assessment of VSA.

Targeted assessments are essential for the evaluation of individual abilities. However, assessments are often carried out in very artificial settings that are far from everyday life play situations. Using a tablet-based app, which has already been shown to maintain young children’s interest over a longer period (Pazouki et al., 2018), may help to reduce stress in assessment situations. Thus, children’s abilities may be assessed in a more playful manner (Zaranis and Valla, 2017), most probably facilitating the assessment process. Furthermore, features implemented in MaGrid, such as its language neutrality or built-in logging- and monitoring-system, may be assumed to be very promising for a fair and simplified data acquisition and monitoring of developmental processes.

In the present study and based on the above-mentioned assumptions, we modified the functionality of MaGrid so that it could be used for the assessment of VSA in kindergarten children. To this end, we chose six tasks of MaGrid, which were most closely related to the tasks Newcombe and Shipley (2015) associated with specific VSA according to their taxonomy: Two tasks each were assigned to assess intrinsic-static, intrinsic-dynamic, and extrinsic-static VSA. Please note that extrinsic-dynamic VSA (i.e., perspective taking) cannot be assessed using MaGrid because the app does not include respective tasks (see Frick et al., 2014). Therefore, we did not consider extrinsic-dynamic VSA in this study.

Using the six tasks, we evaluated whether the selected tasks conform to the taxonomy of Newcombe and Shipley (2015) applying a CFA approach (cf. Mix et al., 2018, for a similar approach). The CFA approach seems to be well suited to evaluate the structural predictions in the taxonomy of VSA by testing the fit of theoretically specified models against each other. We further investigated whether MaGrid can detect age differences in the development of VSA between three age groups of kindergarten children (youngest group: 48–58 months, intermediate group: 59–67 months, oldest group: > 68 months).

Our hypotheses were as follows: First, we expected the assignment of tasks to the categories of VSA according to the taxonomy of VSA by Newcombe and Shipley (2015) to be reflected by our empirical data as evaluated in the CFA approach. Second, provided that the visual-spatial tasks implemented in MaGrid are sensitive to reflect the hierarchical development of VSA appropriately, we further expected to observe the following specific pattern of task performance: Concerning the latent structure of the VSA according to the 2 × 2 taxonomy, we assumed to find evidence for a hierarchical development of the VSA within and across all three categories. Accordingly, older children should outperform younger children on the respective tasks within each category. Across VSA categories, task performance for intrinsic-static VSA should be better than task performance for intrinsic-dynamic VSA, which should be more pronounced than task performance in extrinsic-static VSA in all groups of children.

## Methods

### Participants

Eighty-six children from four different kindergartens in the state of Baden-Wuerttemberg (Germany) participated in the study. Two children were excluded during data collection due to insufficient German language skills. Finally, data of 84 children (39 girls, mean age: *M* = 63.18 months, *SD* = 8.26 months (range 49–78 months) were included. The parents of 78 children reported that their child had German nationality. Furthermore, 56 children stated that they had experiences with tablet devices regularly.

Written informed consent was obtained from parents prior to the study besides children’s verbal assent before the actual assessment. All children received a small present (e.g., a pencil and a pixie book) for their participation. The study was approved by the local ethics committee (LEK 2018/043).

### Procedure

Data were collected in at least two individual testing sessions lasting ∼40 min. Testing sessions took place in a quiet and well-lit room in the respective kindergartens. Before the testing, all children were familiarized with the MaGrid app in two different ways: First, children could try out the handling of the app by playing around in the “Freeplay” mode (cf. Pazouki et al., 2018). Second, children were instructed by a tutorial video, which preceded each task and showed a visual example of solving an instance of the selected task without verbal instructions. For the assessment, we used a termination criterion to avoid repeated experiences of failure and terminated the task when a child made more than three errors in a row.

### Materials

#### MaGrid Tasks

To assess children’s VSA, we used an adapted version of MaGrid. Adaption involved several changes to the training version of the app. For example, children did not receive any feedback on their provided solutions and could only submit one solution for each item, regardless of whether they found the correct solution or not. In addition, the order of items for each task was fixed. In all tasks, items increased in task difficulty over the course of testing in order to induce variability between the tested age groups.

In the present study, we were interested in children’s task performance as assessed by overall correctness in each task. To this end, an item was evaluated dichotomously as either correct or incorrect (i.e., data), resulting in a sum score for each task assessed.

#### Intrinsic-Static VSA

To assess children’s intrinsic-static VSA, we used the tasks *Find forms* and *Close forms* of the MaGrid app (Pazouki et al., 2018). For the task *Find forms*, children were supposed to select a specific geometric form (e.g., a triangle or a rectangle), which was given in a booklet, from different distracting forms (see Figure 1A, in the top row) by touch-typing. This task included 16 items. In order to increase task difficulty, the number of distractors continuously increased during the task. As the number of distractors on the tablet increases, the size of the forms needed to be decreased in order to fit all forms on the display. Consequently, the size of the target form in the booklet and forms on the display varied for the more difficult trials (i.e., in 6 of 16 items). Therefore, it was explained to participating children beforehand that the size of forms in the booklet and on the tablet may differ in some trials. However, for solving the task, the shape of the form is important and not its size on the display. As *Find forms* relies basically on static pattern recognition, we assumed that the depiction of forms in different sizes should not significantly affect children’s performance in intrinsic-static VSA as one would expect for active scaling processes in intrinsic-dynamic VSA.

For the task *Close forms*, a booklet was also required. The booklet showed a target form. The same form but with missing lines was displayed on the tablet in a grid. Children were asked to complete the form by drawing the missing line with their index finger (see Figure 1A, in the bottom row). This task also consisted of 16 items. The difficulty was increased by eliminating more lines from the given forms. In addition, the corners of a form were no longer displayed, requiring the children to create new corners to complete the forms instead of just connecting two dots in a straight line.

#### Intrinsic-Dynamic VSA

To assess children’s intrinsic-dynamic VSA, we used the MaGrid tasks *Rotation* and *Tangram* (cf. Pazouki et al., 2018). For the *Rotation* task, children were asked to align the given form according to the orientation depicted in the booklet (see Figure 1B, in the top row). To this end, children were supposed to use two rotary buttons. Sixteen items were assessed. In the more difficult trials, form configuration was more specific requiring advanced visual-spatial perception.

The *Tangram* task required children to assemble various geometric forms according to a given configuration in the booklet. The forms to be assembled were presented in a random position on the tablet (see Figure 1B, in the bottom row). Children had to use their fingers to select a form and drag it to the correct position in relation to the other forms. Motor requirements for *Tangram* were comparably medium. *Tangram* comprises 14 items, with to-be-built configurations becoming more complex in later trials. An item was only considered to be solved correctly (and thus awarded 1 point) when all components of the form were correctly assembled (see Verdine et al., 2017, for a discussion of different coding strategies and performance on a similar spatial assembly task).

#### Extrinsic-Static VSA

To assess children’s extrinsic-static VSA, we used the MaGrid tasks *Reproduce forms* I and II (cf. Pazouki et al., 2018). In the MaGrid task *Reproduce forms I* children had to reproduce (i.e., draw) a given geometric form in the grid of the app according to the form depicted in the booklet (see Figure 1C, in the top row). In sum, 25 items were assessed. The number of the given forms as well as their complexity varied between the easy and the more difficult trials.

The MaGrid task *Reproduce forms II* only differed slightly from the *Reproduce form I.* Instead of in a booklet, the target form was shown on the tablet itself in a specific position in the grid. Children were not only required to copy the given form, but they also had to reproduce the correct position in the grid (see Figure 1C, in the bottom row), and thus adhere to the reference frame. This task comprised 16 items. Again, more difficult tasks varied from easy tasks by using more complex forms.

The motor component for both tasks was rather high, compared to the *Tangram* task, because children had to draw on the tablet in order to copy the figure. Again, an item was only considered to be solved correctly (and awarded 1 point) when the entire form was copied correctly.

### Data Analysis

#### Confirmatory Factor-Analysis – Structure of Early VSA

To evaluate the taxonomy of VSA suggested by Newcombe and Shipley (2015) on our data, we conducted a CFA. In the CFA, we opted on an inclusive strategy. That is, we included as many indicators per factor as possible to compensate for the relatively small sample (as recommended by e.g., Marsh et al., 1998). Items of the *Find forms* and *Close forms* tasks were considered to assess intrinsic-static VSA. Items of the tasks *Rotation* and *Tangram* were classified as assessing intrinsic-dynamic VSA. Items of the two *Reproduce forms* tasks were considered assessing extrinsic-static VSA. As all items were coded binary (i.e., correct: 1, incorrect: 0) we used the Weighted Least Squares Means and Variances (WLSMV) adjusted estimator (e.g., Li, 2016). We considered Root Mean Square Error of Approximation (RMSEA), Comparative Fit Index (CFI), and Tucker-Lewis Index (TLI) to evaluate model fit, with RMSEA < 0.05, CFI > 0.95, and TLI > 0.95 as cut-off criteria for a well-fitting model (Hu and Bentler, 1999). All analyses were performed in Mplus Version 8.0 (Muthén and Muthen, 2017) and SPSS (IBM^®^, SPSS Statistics, Version 25).

#### Hierarchical Development of VSA

To evaluate whether children’s VSA developed hierarchically, we formed three different sub-groups according to children’s age (youngest, intermediate and oldest age-group). The threshold for the oldest group was chosen because these children were old enough to enter school according to the education Act for Baden-Württemberg {Schulgesetz für Baden-Württemberg [SchG, 1983, §73 (1)]}. The second threshold was chosen to form two additional groups of similar sizes (see Table 1). We, therefore, assigned 27 children to the group of youngest children (i.e., 48–58 months old), 26 children were assigned to the intermediate group (i.e., 59–67 months old), and 31 children were assigned to the group of oldest children (i.e., 68–78 months old). This allowed us to investigate children’s intrinsic-static, intrinsic-dynamic and extrinsic-static VSA separately for each age-group.

**TABLE 1:** Sub-groups according to children’s age.

**Age-group**	**Age (months)**	***M (SD)***	**N**	**Gender (m:f)**
Youngest	48–58	53.33 (2.96)	27	12:15
Intermediate	59–67	63.19 (2.67)	26	17:9
Oldest	>68	71.74 (3.47)	31	16:15

To test the hierarchical development of VSA in young children, we conducted both *t*-tests in order to investigate overall differences in children’s task performance and a MANOVA evaluating the influence of age on the different categories. VSA was measured by the mean scores of correct answers for a task, with two tasks representing one ability (e.g., the intrinsic-static ability is measured by the mean score of the correct answers for *Find forms* and *Close forms*). As 56 children had prior tablet experience, we analyzed whether this experience moderated performance across tasks using the SPSS-macro PROCESS (Hayes, 2012).

The significance level was set to *p* ≤ 0.05 for all analyses. Effect sizes are reported as η^2^*_*p*_* (medium effect ≥ 0.06, large effect ≥ 0.14, according to the recommendations of Cohen (1969, see also Richardson, 2011). Bonferroni-corrected pairwise comparisons followed-up the univariate analyses to specify significant group differences.

## Results

In total, data of 84 children entered the analyses. Table 2 provides descriptive information regarding the group mean performance of the six selected MaGrid tasks. As all items were binary coded, the mean scores of the tasks indicate the percentage of correctly solved items for each task.

**TABLE 2:** Task performance for each age group (mean correct and standard deviation).

**Task**	**Youngest**	**Intermediate**	**Oldest**
	***Mean* (*SD*)**	***Mean* (*SD*)**	***Mean* (*SD*)**
Find forms	0.85 (0.15)	0.91 (0.09)	0.90 (0.08)
Close forms	0.73 (0.15)	0.80 (0.19)	0.87 (0.12)
Rotation	0.82 (0.17)	0.89 (0.15)	0.93 (0.09)
Tangram	0.36 (0.27)	0.57 (0.22)	0.71 (0.18)
Reproduce forms I	0.08 (0.14)	0.20 (0.21)	0.32 (0.27)
Reproduce forms II	0.46 (0.38)	0.76 (0.22)	0.82 (0.19)

We also looked at the correlations between tasks and found significant correlations between all tasks. Table 3 indicated that most correlations were moderate to high (Cohen, 1988) except for the correlation between *Find forms* and *Reproduce forms I*.

**TABLE 3:** (Pearson) correlations between MaGrid tasks.

	***CF***	***RO***	***T***	***RI***	***R II***
Find forms	*r* = 0.32*	*r* = 0.46*	*r* = 0.39*	*r* = 0.23*	*r* = 0.41*
Close forms		*r* = 0.44*	*r* = 0.63*	*r* = 0.48*	*r* = 0.58*
Rotation			*r* = 0.51*	*r* = 0.38*	*r* = 0.48*
Tangram				*r* = 0.61*	*r* = 0.77*
Reproduce forms I					*r* = 0.54*

All correlations are significant at (p < 0.05), as indicated by the asterisk (*) with CF, Close Forms; RO, Rotation; T, Tangram; RI, Reproduce Forms I; R II, Reproduce Forms II.

### Confirmatory Factor Analysis: Structure of Early VSA

**FIGURE 2:**
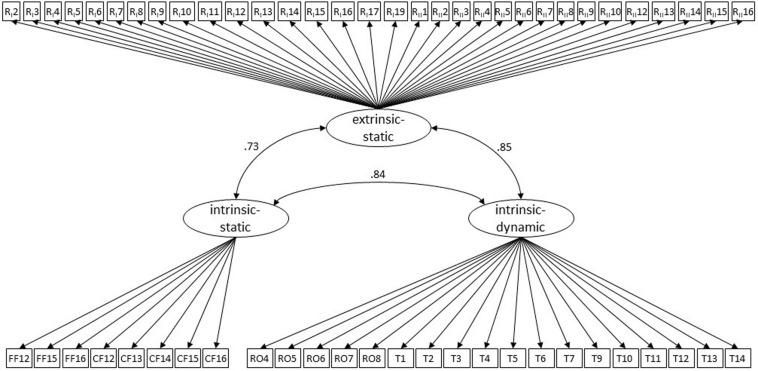
Confirmatory factor analysis – latent structure of early VSA. The figure shows all items that were considered in the analysis. The three latent factors (i.e., intrinsic-static, intrinsic-dynamic, and extrinsic-static VSA) are derived from the 2 by 2 taxonomy of Newcombe and Shipley (2015). The figure depicts also correlations (all *p* < 0.001) among the three latent factors. To increase readability, factor-loadings and error terms of items are not displayed. Intrinsic-static VSA were measured by items of the *Find forms* and *Close forms* tasks (i.e., FF and CF), intrinsic-dynamic VSA by items of the *Rotation* and *Tangram* tasks (i.e., RO and T), and extrinsic-static VSA by items of the *Reproduce forms I* and *II* tasks (i.e., RI and RII). See text and Table 5 for more details.

We first analyzed the relative frequencies of correct and incorrect solutions in all 103 items. Items with low variance (i.e., items that were correctly or incorrectly solved by at least 90% of the children) were excluded as they did not entail sufficient information for model estimation (i.e., 44 items). Based on the remaining 59 items, we specified a three-factor model. In this model, intrinsic-static VSA were indicated by items from the *Find forms* and *Close forms* tasks (9 items in total). Intrinsic-dynamic VSA were indicated by items from the *Rotation* and *Tangram* tasks (18 items in total). Extrinsic-static VSA were reflected by items from the two *Reproduce forms* tasks (32 items in total). The model provided a good fit to the data, χ^2^_(1649)_ = 1771.64, *p* = 0.020.02, RMSEA = 0.03, 90% CI: [0.014; 0.041], CFI = 0.98, TLI = 0.98. One additional item considered to reflect intrinsic-static VSA was dropped due to non-significant factor loading. However, model fit did not change substantially, χ^2^_(1592)_ = 1717.68, *p* = 0.01, RMSEA = 0.03 90% CI: [0.015; 0.041], CFI = 0.98, TLI = 0.98. Taken together, these results indicate that the hypothesized three-factor structure according to Newcombe and Shipley (2015) was substantiated by the current data for kindergarten children. Item descriptions and factor loadings are presented in Table 4. Moreover, intrinsic-static VSA were found to be highly correlated with intrinsic-dynamic VSA (*r* = 0.84, *p* < 0.001). Similar high correlations were observed for intrinsic-static and extrinsic-static VSA (*r* = 0.73, *p* < 0.001) as well as for intrinsic-dynamic and extrinsic-static VSA (*r* = 0.85, *p* < 0.001). The final model is shown in Figure 2.

**TABLE 4:** Descriptive statistics and factor loadings for items from the MaGrid app.

**Factor**	**Item**	**% Correct**	**Factor loading**	**Factor**	**Item**	**% Correct**	**Factor loading**	**Factor**	**Item**	**% Correct**	**Factor loading**
IS	FF12	0.488	0.765	ID	RO4	0.774	0.495	ES	RI3	0.357	0.854
IS	FF15	0.655	0.691	ID	RO5	0.679	0.821	ES	RI4	0.440	0.777
IS	FF16	0.798	0.605	ID	RO6	0.750	0.785	ES	RI5	0.429	0.869
IS	CF12	0.583	0.697	ID	RO7	0.798	0.552	ES	RI6	0.119	0.646
IS	CF13	0.405	0.824	ID	RO8	0.631	0.748	ES	RI7	0.357	0.854
IS	CF14	0.476	0.858	ID	T1	0.667	0.464	ES	RI8	0.226	0.845
IS	CF15	0.381	0.828	ID	T2	0.679	0.697	ES	RI9	0.238	0.895
IS	CF16	0.512	0.977	ID	T3	0.726	0.902	ES	RI10	0.393	0.843
				ID	T4	0.798	0.823	ES	RI11	0.179	0.716
				ID	T5	0.857	0.905	ES	RI12	0.214	0.874
				ID	T6	0.238	0.563	ES	RI13	0.143	0.701
				ID	T7	0.345	0.609	ES	RI14	0.333	0.940
				ID	T9	0.631	0.802	ES	RI15	0.274	0.959
				ID	T10	0.655	0.948	ES	RI16	0.238	0.935
				ID	T11	0.345	0.754	ES	RI17	0.214	0.898
				ID	T12	0.702	0.621	ES	RI19	0.131	0.840
				ID	T13	0.464	0.745	ES	RII1	0.690	0.775
				ID	T14	0.548	0.800	ES	RII2	0.571	0.553
								ES	RII3	0.845	0.907
								ES	RII4	0.571	0.673
								ES	RII5	0.750	0.902
								ES	RII6	0.810	0.957
								ES	RII7	0.702	0.909
								ES	RII8	0.786	0.878
								ES	RII9	0.810	0.983
								ES	RII10	0.524	0.746
								ES	RII12	0.643	0.797
								ES	RII13	0.762	0.917
								ES	RII14	0.667	0.841
								ES	RII15	0.369	0.668
								ES	RII16	0.607	0.871

**IS, intrinsic-static VSA; ID, intrinsic-dynamic VSA; ES, extrinsic-static VSA.**

### Hierarchical Development of Early VSA

Although not at the heart of the current research question, we first checked for overall differences in children’s task performance on the three VSA. As indicated by Bonferroni-corrected *t*-tests, task performance was significantly better for intrinsic-static VSA (*M* = 0.85, *SD* = 0.11) than for both intrinsic-dynamic VSA [*M* = 0.73, *SD* = 0.18, *t*_(83)_ = 8.55, *p* < 0.001] and extrinsic-static VSA [*M* = 0.39, *SD* = 0.24, *t*_(83)_ = 22.01, *p* < 0.001]. Moreover, the difference between intrinsic-dynamic and extrinsic-static VSA was also significant [*t*_(83)_ = 20.10, *p* < 0.001].

Due to the unequal distribution of boys and girls in the intermediate group, preliminary analysis by means of a MANCOVA considering sex as the covariate were conducted. There was no significant influence of the covariate sex overall [Pillai-Trace = 0.031, *F*_(3,78)_ = 0.820, *p* = 0.487] as well as for the VSA categories as indicated by univariate follow-up analyses: intrinsic-static: [*F*_(1,80)_ = 0.556, *p* = 0.458; intrinsic-dynamic: *F*_(1,80)_ = 0.012, *p* = 0.914; extrinsic-static: *F*_(1,80)_ = 0.807, *p* = 0.372]. Based on these results, we are confident that the unequal distribution of boys and girls in the intermediate group did not drive our results.

To gain a better understanding of the hierarchical development of VSA, we conducted a MANOVA that indicated a significant age effect for VSA [Pillai-Trace = 0.30, *F*_(6, 160)_ = 4.78, *p* < 0.001, η*^2^_*part.*_* = 0.99, see Table 5].

**TABLE 5:** Task performance for the different age groups.

**Categories**	**Tasks**	**Age group**	***M***	***SD***	**N**	***F***	***p***	**η*^2^_*part.*_***
Intrinsic-static	Find forms Close forms	Youngest	0.79	0.13	27	5.81	0.004	0.13
		Intermediate	0.86	0.11	26			
		Oldest	0.89	0.07	31			
Intrinsic-dynamic	Rotation Tangram	Youngest	0.61	0.19	27	14.48	0.000	0.26
		Intermediate	0.74	0.16	26			
		Oldest	0.82	0.11	31			
Extrinsic-static	Reproduce forms I Reproduce forms II	Youngest	0.23	0.21	27	14.51	0.000	0.26
		Intermediate	0.42	0.19	26			
		Oldest	0.52	0.21	31			

**The table depicts mean correct (SD) for each age group, the number of children in each group and the test statistics for each ability.**

Follow-up univariate analyses indicated that there was a significant medium sized age effect for intrinsic-static VSA [*F*_(2, 81)_ = 5.81, *p* = 0.004, η*^2^_*part.*_* = 0.13]. Bonferroni-corrected pairwise comparisons showed a significant difference between the youngest and oldest group only (*p* = 0.003).

For intrinsic-dynamic VSA, univariate analysis revealed a similar significant age effect with a large effect size [*F*_(2, 81)_ = 14.48, *p* < 0.001, η*^2^_*part.*_* = 0.26]. Bonferroni-corrected pairwise comparisons indicated that the task performance of children in the youngest and oldest group (*p* < 0.001) differed significantly. The same applied to children in the youngest and intermediate group (*p* = 0.008).

**FIGURE 3:**
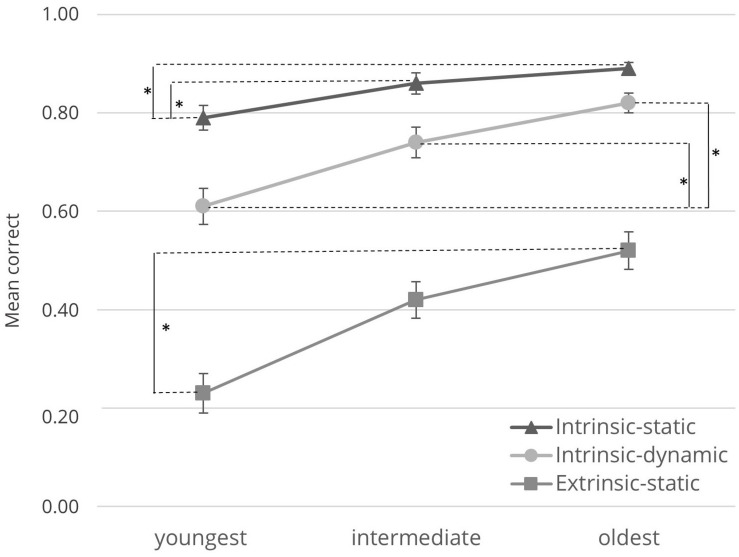
Task performance for each age group. *M* (Mean Correct) for all three sub-groups for the tested abilities (black, intrinsic-static VSA; light-gray, intrinsic-dynamic VSA; gray, extrinsic-static VSA). Error bars reflect 1 *SE*. Significant differences with *p* < 0.05, as indicated by the asterisk (*).

Finally, for extrinsic-static VSA, univariate analysis indicated a significant age effect with a large effect size [*F*_(2, 81)_ = 14.51, *p* < 0.001, η*^2^_*part.*_* = 0.26]. Again, Bonferroni-corrected pairwise comparisons indicated significant age differences between the youngest and intermediate group (*p* = 0.003) and the youngest and oldest group (*p* < 0.001). Figure 3 depicts children’s task performance for each category of VSA. The figure visualizes that group differences exist only between the youngest and the intermediate group for intrinsic-dynamic and extrinsic-static VSA, or for the youngest and oldest group (all VSA). Crucially, no differences were observed between the intermediate and oldest group.

Results of a moderation analysis further indicated that children’s prior experience with tablets did not moderate performance in intrinsic-static VSA, β = –0.04, *p* = 0.137), intrinsic-dynamic VSA (β = –0.04, *p* = 0.318), nor extrinsic-static VSA (β = 0.003, *p* = 0.956). These findings indicate that children’s prior experience with tablets did not moderate the relationship between age and performance on the assessed VSA significantly.

## Discussion

The present study aimed at evaluating the hierarchical development of VSA from both a theoretical and a behavioral perspective. For this aim, we selected six different visual-spatial tasks of the tablet-based app MaGrid (Cornu et al., 2017; Pazouki et al., 2018): two tasks each reflecting the three categories intrinsic-static, intrinsic-dynamic, and extrinsic-static VSA of the 2 by 2 taxonomy of Newcombe and Shipley (2015).

Additionally, we adapted the functionality of MaGrid to use it for assessment purposes. Uttal et al. (2013) claimed VSA to be malleable at an early age. Therefore, accurate and reliable assessment tools are essential to both measure training success and to understand the latent structure underlying the development of VSA.

Results of the CFA indicated that the selected visual-spatial tasks reflected the respective VSA according to the taxonomy of Newcombe and Shipley (2015). Behavioral results showed that MaGrid is sensitive to detect expected age-related differences in performance between younger and older kindergarten children. In the following, we will discuss these findings in more detail beginning with the latent structure of VSA before turning to the discussion of MaGrid as an assessment tool for the development of VSA.

### Latent Structure of VSA According to the 2 by 2 Taxonomy

Our CFA evaluating the structure of VSA according to the 2 by 2 taxonomy of Newcombe and Shipley (2015) indicated a good model fit for the three-factor solution reflecting the three categories of VSA of interest, this means, (i) intrinsic-static, (ii) intrinsic-dynamic, and (iii) extrinsic-static VSA. Factor loadings of all items were at an acceptable level (≥ ∼0.5) alongside with a good overall fit of the model to the empirical data. CFA results suggest that the selected MaGrid tasks can be conceptualized in terms of the three (out of four) VSA as proposed by the taxonomy of Newcombe and Shipley (2015).

As regards theoretical considerations, it is important to note that we needed to exclude some items for the CFA due to insufficient variance in these items: This affected the first items of the tasks assessing the intrinsic-static (i.e., *Find forms* and *Close forms*) and the intrinsic-dynamic VSA (i.e., *Rotation* and *Tangram*). Exclusion of the first (i.e., easy) item suggests that these items may have been too easy for most children of our sample. This is in line with the observed near ceiling effects which we found for intrinsic-static VSA. Interestingly, the exclusion also affected the last items of the tasks assessing extrinsic-static VSA (i.e., *Reproduce forms I* and *Reproduce forms II).* Here, item exclusion suggests that these items may have been rather difficult for the children of our sample. Crucially, item exclusion should not negatively affect our interpretation of results. Even for the reduced number of items representing intrinsic-static VSA the statistical requirements for a just-identified factor were fulfilled, because factor loadings can be estimated independent of any particular item score (Brown, 2014).

However, analysis of response times may help to solve this issue in future studies. For instance, response times have been found to reflect specific effects of numerical processing related to visual-spatial concepts (i.e., the SNARC effect Dehaene et al., 1993). Moreover, response times and accuracy can be combined, for instance as a rate correct score (Woltz and Was, 2006), which then reflects the number of correct answers per second. It would be desirable to further pursue these avenues in future studies.

Furthermore, CFA results provided further evidence with respect to the assumptions of a hierarchical structure of the 2 by 2 taxonomy of Newcombe and Shipley (2015). CFA showed similarly high correlations between the three different factors (> 0.73). These correlations suggest that despite the division into different categories, the three VSA assessed in the current study (i.e., intrinsic-static, intrinsic-dynamic and extrinsic-static) can hardly be considered to reflect distinct constructs. Instead, they seem to represent most probably hierarchically developing VSA, and thus, help to specify the hierarchical structure of VSA, for which literature is still lacking a common definition (Eliot and Smith, 1983; Carroll, 1993; Newcombe and Shipley, 2015; Mix et al., 2016). Providing evidence of a hierarchical development and/or latent structure of VSA in the taxonomy by Newcombe and Shipley (2015) seems a major challenge for at least two main reasons: first, it may be the case that children at the age of 3 cannot solve a visual-spatial task in an assessment while they are able to solve the task during playing, in which they can master the necessary perception and action steps (Newcombe, 2018). Second, it may be that the same task requires more than one VSA to be solved (Mix et al., 2018). According to the findings of Verdine et al. (2017), spatial assembly tasks, such as the *Tangram* task are complex activities involving more than one visual-spatial ability. In the *Tangram* task, the presented form and its components need to be encoded first (i.e., requiring intrinsic-static VSA) before components need to be moved to the right position to assemble the entire form (i.e., requiring intrinsic-dynamic VSA). Both issues illustrate that theoretical assumptions of an ability and actual behavior when applying this ability do not always correspond perfectly.

### MaGrid as an Age-Sensitive Assessment Tool

On the behavioral level, we observed significant age effects for all three categories (i.e., intrinsic-static, intrinsic-dynamic and extrinsic static), which was in line with our hypothesis. In all categories, we found significant differences in task performance between 4-years old (i.e., youngest group) and 6-years old (i.e., oldest group) children. Additionally, we observed significant differences between 4- and 5-years old (i.e., intermediate group) children in intrinsic-dynamic and extrinsic-static VSA. The performance of the 5- and 6-years old children did not differ significantly in any category. These results suggest MaGrid to be sensitive enough to differentiate between VSA of 4- and 6-years old children. Furthermore, the tasks assessing intrinsic-static VSA might have been too easy for children of all age groups. This might explain why only intrinsic-dynamic and extrinsic-static VSA tasks differentiated successfully between 4- and 5-years old children. However, for the latter two categories, we did not observe significant differences between the performance of 5- and 6-years old children which was contrary to our expectations. This finding might be explained by the fact that MaGrid might either not be sensitive enough to differentiate between the two age groups or the development level of the two age groups may have been too similar.

In addition to these observations, performance was higher for intrinsic-static tasks than for extrinsic-static tasks substantiating the hierarchical order of the development of these categories. This finding is particularly evident from the ceiling effects for the group of 6-years-old children for the task *Find forms*. This task requires elaborate shape recognition and abstract representation of the respective forms (i.e., intrinsic-static VSA). The task *Close forms*, which requires additional visual motor integration (Pazouki et al., 2018), demands the coordination between perceived visual input and motor output to complete the unfinished objects according to the booklet (Cornu et al., 2017).

In this context, Beery et al. (2010) observed that the development of visual motor integration was closely associated with the development of motor skills in general. In their study, they investigated this development from the ability to copy vertical lines (at the age around 2 years) and circles (at the age around 3 years) to the ability to trace horizontal lines (at the age of 3.5 years) and to connect two dots by a horizontal line (at the age of 4.5 years; Beery et al., 2010). As the youngest children in our study were 4 years and older, it is not surprising that the task *Close forms* was mastered differently well by children of different age groups.

Tasks involving intrinsic-dynamic VSA were observed to be more difficult for younger children resulting in performance differences between age groups. As dynamic VSA involve transforming and manipulating objects, such as the tasks *Tangram* and *Rotation*, they may pose higher cognitive demands. Even though it was observed in 2-year-old children that they are able to solve tasks assessing intrinsic-dynamic VSA sufficiently through perception-action skills (e.g., inserting 3D forms into appropriate slots of a box, Örnkloo and von Hofsten, 2007), this may not necessarily imply generalizability to the tasks as used in the present study (Newcombe, 2018).

Among all tasks we selected from MaGrid to assess intrinsic VSA, the *Tangram* task was the most demanding task as it requires solving visual-spatial problems by categorizing and comparing objects in relation to each other (Lin et al., 2011). Several studies indicated that tangrams inspire shape analysis, integration, and composition of objects as well as logical thinking (e.g., Olkun et al., 2005; Lin et al., 2011), and thus might be considered one of the best methods to enhance geometrical spatial thinking (Verdine et al., 2017). With its medium task difficulty and its potential involvement of other VSA (i.e., considering spatial relations of objects during visual assembly), *Tangram* seemed very suitable for assessing VSA in kindergarten children.

Finally, the most complex and difficult tasks were those assessing extrinsic-static VSA (i.e., *Reproduce forms I* and *II*), for which children of all age groups performed most poorly. The higher task demands manifested in higher variance in performance on the individual items of the tasks. Even 6-years old children in our study did not perform perfectly on these tasks and may thus not have acquired this category of VSA fully yet. This is in line with current findings showing that the understanding of spatial relations between objects and the environment as well as the size and scaling of objects improves with age and individual experiences (Newcombe and Huttenlocher, 2006; Okamoto et al., 2015).

Taken together, behavioral results indicate that basic VSA are acquired early (see Clements, 1998) and improve steadily with increasing age (Uttal et al., 2013; Newcombe et al., 2015; Cornu et al., 2017). The present results reflect that the age-related development of VSA can be measured using MaGrid. Moreover, exclusion of too easy or too difficult items (solved by almost all or no children, respectively) representing intrinsic or extrinsic VSA in the CFA only reflected the results on the behavioral level. Together, both behavioral and factor-analytical results indicated that the theoretically assumed development of VSA can be found both in the taxonomy of Newcombe and Shipley (2015) and empirically in the current data. This corroborates our theoretical understanding of the structure of VSA and their development. Although some tasks turned out to be more sensitive than others, the overall pattern of results with significant age differences for all VSA assessed corroborates the claim that kindergarten age seems central for the development of VSA (Newcombe and Frick, 2010; Cornu et al., 2017).

### Limitations

When interpreting the results of the current study, some limiting aspects need to be considered. First, even though CFA models converged, our sample size is smaller than the commonly suggested lower bounds for conducting CFA of at least *N* = 100 (e.g., Anderson and Gerbing, 1988). However, there is also evidence that models can be meaningfully estimated with smaller samples. In particular, it seems that a large number of indicators per latent factor, high factor loadings, and high intercorrelations among factors may substantially decrease the required sample size (e.g., Marsh et al., 1998; Wolf et al., 2013). Given that all these aspects applied to the present data, it seems rather unlikely that sample size is a source of bias in the analyses.

Moreover, it has to be noted that several items had little to no variance and needed to be excluded from the CFA. Lack of variance was primarily caused by items that were solved correctly by almost all or no children. For future studies, it would be desirable to use additional items of medium difficulty as well as items that can differentiate also in a lower and upper ability range.

Finally, it needs to be considered that the study was cross-sectional observing VSA in children of different age levels. As such, we did not monitor the intra-individual development of children longitudinally, which means that the interpretation of developmental aspects needs to be done cautiously. Nevertheless, we think that interpretations of the development of VSA seem warranted as the present results correspond closely to previous findings (e.g., Uttal et al., 2013; Okamoto et al., 2015). Yet, future longitudinal studies would be desirable to investigate the development of (the latent structure of) VSA in more detail.

## Conclusion

In the current study, we investigated the development and structure of VSA in kindergarten children (i.e., aged 4–6 years) using a theoretical and a behavioral approach. On the theoretical level, and based on the CFA, we found evidence to assume the latent structure of VSA as proposed in the 2 by 2 taxonomy of Newcombe and Shipley as valid (2015; but see Mix et al., 2018 for contradicting findings), and may indicated hierarchical development. On the behavioral level, we found that the development of VSA was captured by MaGrid as reflected by age differences. Moreover, we observed that the selected visual-spatial tasks fit well with the differentiation of intrinsic-static, intrinsic-dynamic, and extrinsic-static categories as proposed by this taxonomy. Thereby, these results help specify the theoretical concept of early VSA.

To conclude, the present study contributes to the literature by evaluating and validating a tablet-based assessment of early VSA. On a more theoretical level, the current study indicates that MaGrid assesses VSA on the sound theoretical basis of the taxonomy of Newcombe and Shipley (2015). On the behavioral level, the MaGrid app was found to successfully reflect individual differences in VSA in kindergarten children. In this sense, tablet-based assessments included in this educational app seem to be suitable not only for training but also for assessing VSA.

## Data Availability Statement

All datasets generated for this study are included in the article/Supplementary Material.

## Ethics Statement

The studies involving human participants were reviewed and approved by Local ethic commitee of the Leibniz-Institut für Wissensmedien (LEK 2018/043). Written informed consent to participate in this study was provided by the participants’ legal guardian/next of kin.

## Author Contributions

TP programmed the app for diagnostic purposes. SJ, SR, VC, CS, and KM designed the study. SJ conducted the experiment. SJ, AM, and DB analyzed the data. SJ, AM, DB, and TP wrote the original draft of the manuscript. SJ, AM, and KM reviewed and approved the final version of the manuscript. All authors contributed to the conceptualization of the study.

## Conflict of Interest

The authors declare that the research was conducted in the absence of any commercial or financial relationships that could be construed as a potential conflict of interest.

## Supplementary Material

DATA SHEETS S1, S2 Raw data.
